# The Effects of Variable, Intermittent, and Continuous Negative Pressure Wound Therapy, Using Foam or Gauze, on Wound Contraction, Granulation Tissue Formation, and Ingrowth Into the Wound Filler

**Published:** 2012-01-24

**Authors:** Malin Malmsjö, Lotta Gustafsson, Sandra Lindstedt, Bodil Gesslein, Richard Ingemansson

**Affiliations:** Departments of ^a^Ophthalmology; ^b^Cardiothoracic Surgery, Lund University and Skåne University Hospital, Lund, Sweden

## Abstract

**Objective:** Negative pressure wound therapy (NPWT) is commonly used in the continuous mode. Intermittent pressure therapy (IPT) results in faster wound healing, but it often causes pain. Variable pressure therapy (VPT) has therefore been introduced to provide a smooth transition between 2 different pressure environments, thereby maintaining the negative pressure environment throughout the therapy. The aim of the present study was to examine the effects of IPT and VPT on granulation tissue formation. **Method:** A peripheral wound in a porcine model was treated for 72 hours with continuous NPWT (-80 mm Hg), IPT (0 to -80 mm Hg), or VPT (-10 to -80 mm Hg), using foam or gauze as wound filler. Wound contraction and force to remove the wound filler were measured. Biopsies from the wound bed were examined histologically for granulation tissue formation. **Results:** Intermittent pressure therapy and VPT produced similar results. Wound contraction was more pronounced following IPT and VPT than continuous NPWT. Intermittent pressure therapy and VPT resulted in the formation of more granulation tissue than continuous NPWT. Leukocyte infiltration and tissue disorganization were more prominent after IPT and VPT than after continuous NPWT. Granulation tissue grew into foam but not into gauze, regardless of the mode of negative pressure application, and less force was needed to remove gauze than foam. **Conclusions:** Wound contraction and granulation tissue formation is more pronounced following IPT and VPT than continuous NPWT. Granulation tissue grows into foam but not into gauze. The choice of negative pressure mode and wound filler is crucial in clinical practice to optimize healing while minimizing pain.

The use of negative pressure wound therapy (NPWT) has evolved over the past decade because of its remarkable effects on the healing of chronic and difficult wounds.^1^^,^^2^ It has been shown that NPWT creates a moist environment,^3^ drains exudate,^1^^,^^4^^,^^5^ reduces tissue edema,^6^ contracts the wound edges,^1^^,^^4^^,^^5^ mechanically stimulates the wound bed,^7^^-^9 alters blood flow in the wound edges,^4^^,^^7^^,^^9^^-^11 and stimulates angiogenesis^12^^,^^13^ and the formation of granulation tissue.^4^ Also, NPWT can offer protection against infection, as the wound is sealed. The biological effects of NPWT on the wound bed depend on the type of dressing and the level of negative pressure applied.

The most common mode of negative pressure application is the continuous mode, during which the pressure level is kept constant, for example, at -80 mm Hg. When the negative pressure is repeatedly switched on and off (eg, alternating between 0 and -80 mm Hg), this is called *intermittent pressure therapy* (IPT). This therapy is not often used clinically as the sudden changes in pressure cause the foam to expand and contract repeatedly over the granulation tissue, causing pain. Variable pressure therapy (VPT) has therefore been introduced to provide a smooth transition between 2 different levels of negative pressure (eg, -10 and -80 mm Hg), thereby maintaining the negative pressure environment throughout the therapy.^14^ Figure 1 illustrates the different modes of pressure application. It has been shown that the amount of granulation tissue in the wound bed increases dramatically during IPT.^4^ This may be the result of both mechanical stimulation of the wound bed (a massaging effect) and enhanced blood flow to the wound edges.^15^ The effects of VPT on granulation tissue formation have not yet been examined in a detailed and controlled study.

Today, either polyurethane foam or gauze is used as wound filler in NPWT. The choice of material has considerable influence on the healing process, as can be seen by the amount of granulation tissue and its character. The granulation tissue formed under foam is thick but fragile,^16^^,^^17^ while under gauze it is thinner and more stable.^16^^,^^17^ There is ingrowth of tissue into foam,^18^ but not into gauze.^17^ Therefore, more force is required to remove foam than gauze dressings.^17^ The adherence of foam to the wound bed may lead to material being left in the wound, which may later act as a foreign body. Furthermore, the tearing off of ingrown tissue results in pain during dressing changes.^19^ Krasner^20^ has addressed the problem of pain during dressing changes with foam. The effects of different wound fillers have been studied in detail using continuous NPWT. However, the effects on the adhesion of foam and gauze to the wound bed during IPT or VPT are not known.

The aim of the present study was to examine the effects of IPT and VPT on wound contraction, and granulation tissue formation, including wound bed adhesion and ingrowth into the wound filler, and to compare them with those resulting from continuous NPWT. The study was carried out using a porcine peripheral wound model. The wounds were treated for 72 hours with continuous NPWT (-80 mm Hg), IPT (0 to -80 mm Hg), or VPT (-10 to -80 mm Hg), using foam or gauze.

## MATERIAL AND METHODS

### Animals

Eight healthy domestic pigs of both sexes, with a mean body weight of 70 kg, were fasted overnight with free access to water. The experimental protocol for this study was approved by the Ethics Committee for Animal Research, Lund University, Sweden. All animals received humane care in compliance with the European Convention on Animal Care.

### Anesthesia and surgical procedure

Premedication was performed with an intramuscular injection of xylazine (Rompun vet. 20 mg/mL; Bayer AG, Leverkusen, Germany; 2 mg/kg) mixed with ketamine (Ketaminol vet. 100 mg/mL; Farmaceutici Gellini S.p.A., Aprilia, Italy; 20 mg/kg). Two peripheral veins in the pig's ear were cannulated for induction and maintenance of anesthesia and for fluid administration. Anesthesia was maintained with a continuous infusion of ketamine (Ketaminol vet. 50 mg/mL; Farmaceutici Gellini S.p.A; 0.4–0.6 mg/kg/h). Complete neuromuscular blockade was achieved with a continuous infusion of pancuronium bromide (Pavulon; N.V. Organon, Oss, the Netherlands; 0.3–0.5 mg/kg/h). Fluid loss was compensated for by a continuous infusion of Ringer's acetate at a rate of 200 mL/kg/h for the first 24 hours, followed by 110 mL/h for the remainder of the experiment. The animals received total parenteral nutrition (Kabiven; Fresenius Kabi AB, Uppsala, Sweden). Antibiotics were given once daily as intravenous bolus injections (Streptocillin vet. 250 mg/mL + 200 mg/mL; Boehringer Ingelheim Vetmedica, Malmö, Sweden; 10 mL). The animals were orally intubated with cuffed endotracheal tubes. Mechanical ventilation was established with a Siemens-Elema ventilator (Siemens-Elema AB, Solna, Sweden) in the volume-controlled mode (65% nitrous oxide, 35% oxygen). Ventilatory settings were identical for all animals (respiratory rate, 15 breaths/min; minute ventilation, 12 L/min). A positive end-expiratory pressure of 5 cmH_2_O was applied. A Foley catheter was inserted into the urinary bladder through a suprapubic cystostomy. After the experiments were completed, the animals were euthanized with a lethal dose (60 mmol) of intravenous potassium chloride.

### Wound treatment and study protocol

**Figure 1:**
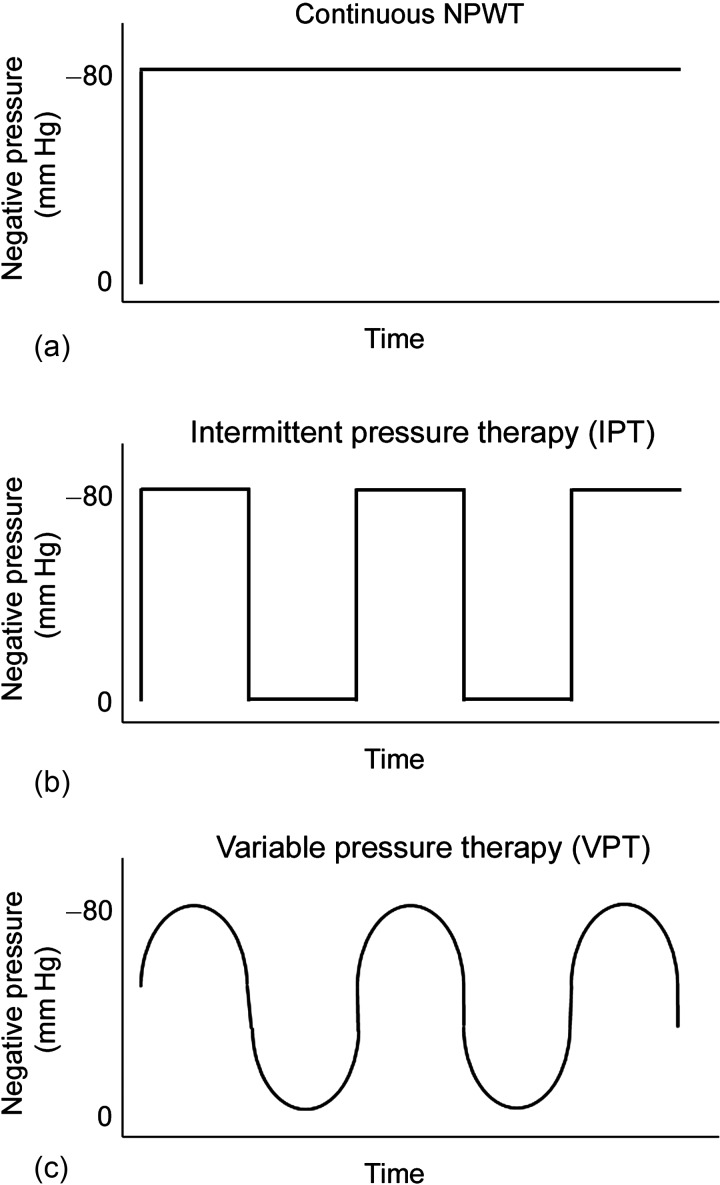
Schematic illustration of the different modes of applying negative pressure. (*a*) In continuous negative pressure wound therapy (NPWT), the pressure is kept constant, for example, at -80 mm Hg. (*b*) In intermittent pressure therapy (IPT), the negative pressure is repeatedly switched on and off (for example, alternating between 0 and -80 mm Hg). (*c*) In variable pressure therapy (VPT), the pressure is varied smoothly between 2 levels (for example, -10 and -80 mm Hg), thus maintaining a negative pressure environment throughout the therapy.

Circular wounds, 6 cm in diameter, extending into the subcutaneous tissue, were created on each pig's back. AMD gauze (Kendall Healthcare, Mansfield, Massachusetts) or black polyurethane foam with an open-pore structure (VAC black GranuFoam, KCI, San Antonio, Texas) was used as wound fillers. The gauze was soaked with saline. AMD gauze contains polyhexamethylene biguanide, which is an antimicrobial agent proven to prevent bacterial colonization within the dressing, and reduce bacterial penetration through the dressing. A drainage tube was inserted into the gauze/foam and connected to a vacuum source (Prospera PRO-III, Prospera Technologies LLC, Fort Worth, Texas). The wound was then sealed with a transparent adhesive drape, which overlapped the wound margins by 10 cm. The wounds were subjected to 72 hours of NPWT without negative pressure (0 mm Hg), continuous NPWT at -80 mm Hg, IPT (0 to -80 mm Hg) or VPT (-10 to -80 mm Hg), using foam or gauze as wound fillers. The exact pressure setting and timing during the therapy is exemplified in Figure 1.

Wound contraction and the force required to remove the wound filler were measured, and sections of biopsies from the wound bed were taken for histological examination (see later).

### Wound contraction

The distance between the wound edges was measured and the wound surface area was calculated. Measurements were performed before the application of negative pressure, immediately after negative pressure was applied (0 hours), every 24 hours, and when negative pressure has been discontinued. The experimental procedure has been described in detail elsewhere.^7^

### Quantity of granulation tissue

The quantity of granulation tissue after NPWT was graded on a scale from 0 to 5 by 2 different surgeons. Grading was performed blinded and separately by each surgeon.

### Force measurements

After 72 hours of NPWT (as described earlier), the adhesive drape covering the wound was cut along the borderline between the tissue and the wound filler, and the drain was removed. The wound filler was attached to a force measurement device and withdrawn at a constant speed of 4 mm/s. The force required to remove the filler from the wound was plotted as a function of time using a computer, and the average force was calculated. The experimental setup has been described in detail previously.^17^

### Histological examination

A strip of the wound filler material (1 × 1 × 2 cm^3^) was sutured onto the bottom of each wound. After discontinuation of NPWT, the strip and the underlying wound bed tissue were excised with a scalpel. The tissue was then treated in 4% paraformaldehyde, dehydrated and embedded in paraffin, and then left overnight. The biopsies were sectioned (4- µm thick), using a rotary microtome HM 355 (ThermoFisher Scientific, Massachusetts), and mounted on glass slides. Prior to hematoxylin-eosin staining, the tissue sections were deparaffinized for 2 × 4 minutes in xylene, 2 × 3 minutes in 99.5% ethanol, and 3 minutes in tap water. The slides were then stained for 12 minutes in Mayer's hematoxylin (Histolab AB, Rockville, Maryland) and rinsed with tap water for 8 minutes, erythrosin (1.5 g in 500 mL H^2^O) for 6 minutes, tap water for 3 minutes, 99.5% ethanol for 2 × 3 minutes and xylene for 4 minutes.

Biopsy sections were evaluated regarding ingrowth into the wound filler and the morphology of the underlying tissue. The following measurements were performed:

1. Ingrowth into the wound filler (µm into the wound filler).

2. Disorganization of the cells in the wound bed, that is, disruption of the contact between the cells and differences in cell size (µm into the tissue).

3. Leukocyte count (leukocytes per µm^2^).

### Calculations and statistics

Calculations were performed using GraphPad 5.0 software (San Diego, California). Statistical analysis was performed using the Mann-Whitney test when comparing 2 groups, and the Kruskal-Wallis test with Dunn's posttest for multiple comparisons when comparing 3 groups or more. Significance was defined as *P* < 0.05. All differences referred to in the text were statistically significant. Results are presented as means ± the standard error of the mean.

## RESULTS

### Wound contraction

**Figure 2:**
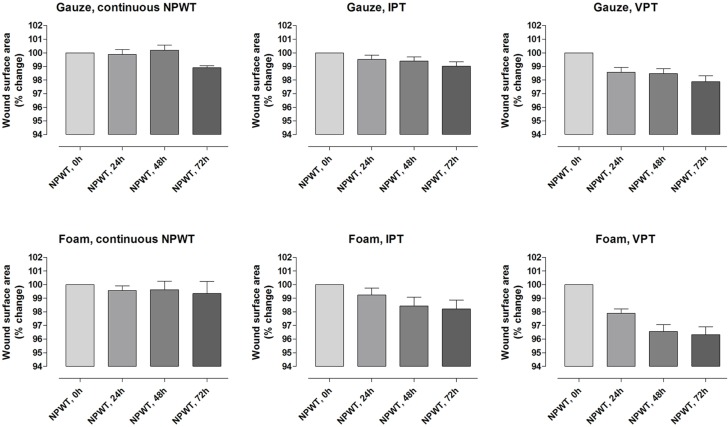
Wound contraction during continuous negative pressure wound therapy (NPWT), intermittent pressure therapy (IPT), and variable pressure therapy (VPT) using gauze or foam. Measurements were performed after 0, 24, 48, and 72 hours of NPWT. The reduction in wound surface area was calculated as a percentage of the baseline area immediately after the negative pressure was applied. Values are presented as means ± standard error of the mean of 8 experiments. Note the gradually decreasing wound area over time with IPT and VPT.

Wound contraction was more pronounced with IPT and VPT than with continuous NPWT. When NPWT was applied in the continuous mode, immediate contraction of the wound was seen, which was maintained for the remaining 72 hours. A different pattern of contraction was seen with IPT and VPT. When negative pressure was applied, there was immediate wound contraction, followed by a gradual decreasing wound area over time. For detailed results, see Figure 2.

### Wound bed morphology

The tissue morphology and the leukocyte infiltration in the wound bed underlying the wound filler were examined histologically.

#### Microdeformation

**Figure 3:**
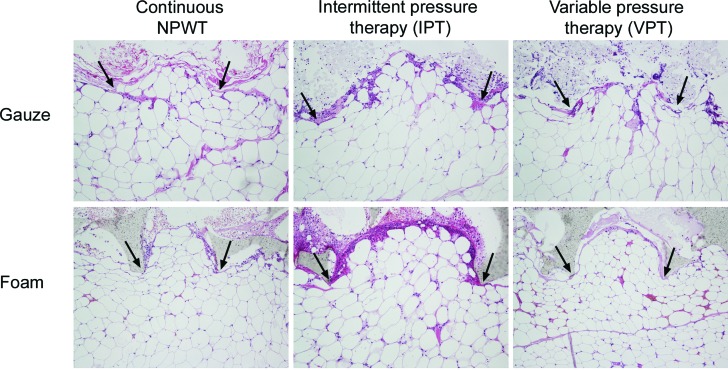
Hematoxylin-eosin stained histological sections of wound biopsy specimens after 72 hours' continuous negative pressure wound therapy (NPWT), intermittent pressure therapy (IPT), or variable pressure therapy (VPT) using gauze or foam. The images show the wound filler (at the top of the images), the interface between the wound filler and the tissue, which was mainly composed of adipocytes (at the bottom of the images). It can be seen that both foam and gauze cause a repeating pattern of wound surface undulations, and the drawing of small tissue blebs into the pores of the foam and the spaces between the threads in the gauze, regardless of the mode of application of negative pressure. The protrusions of wound filler into the tissue are indicated by arrows.

A repeating pattern of wound surface undulations was observed under gauze and foam. This pattern was evident in all wounds, regardless of the mode of negative pressure application (continuous NPWT, IPT, or VPT). The material of the foam and the threads of the gauze compressed the wound bed so that small tissue blebs were drawn into the empty spaces of the foam or the gauze. See Figure 3 for representative examples.

#### Quantity and characteristics of granulation tissue

**Figure 4:**
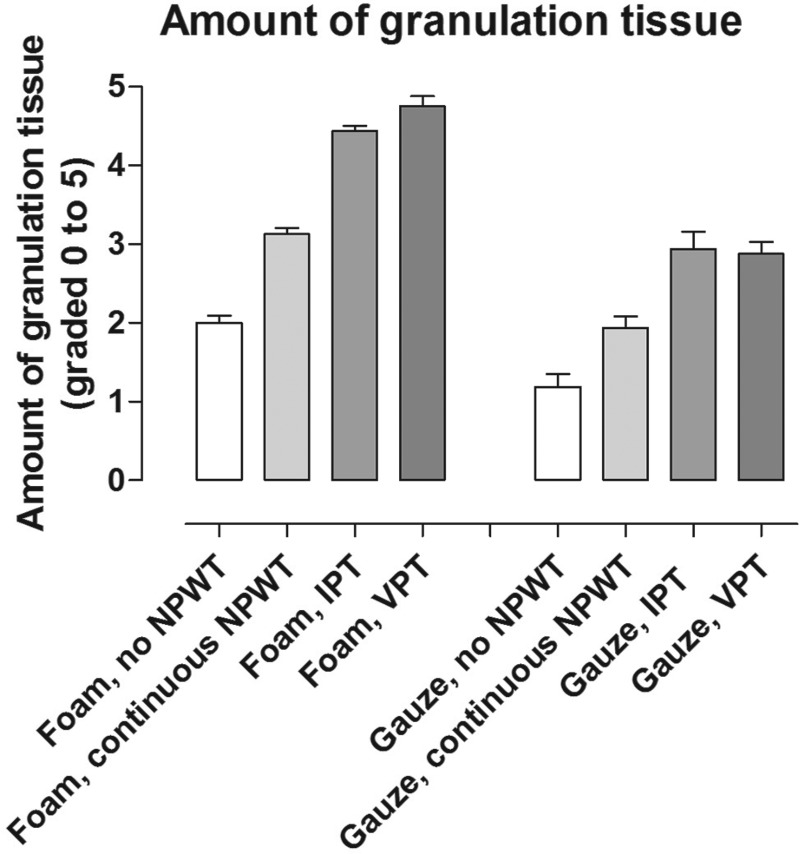
The amount of granulation tissue after dressing application without negative pressure (no negative pressure wound therapy [NPWT]), continuous NPWT, intermittent pressure therapy (IPT), or variable pressure therapy (VPT), using either gauze or foam. The amount of granulation tissue was graded on a scale from 0 to 5 by 2 different surgeons. Results are shown as means ± SEM of 8 experiments. Note that similar amounts of granulation tissue are formed under gauze during IPT or VPT as under foam with continuous NPWT.

**Figure 5:**
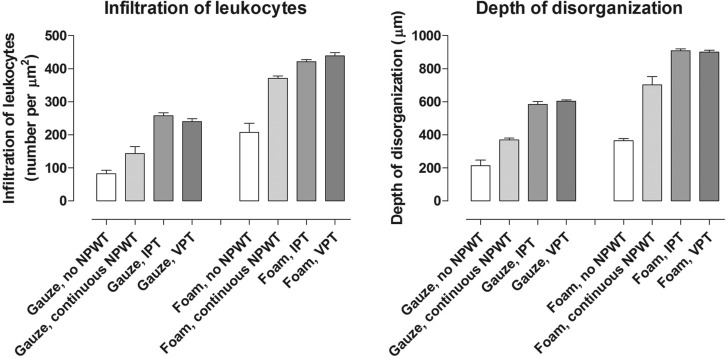
Diagrams showing the number of leukocytes per µm^2^ and the depth of tissue disorganization after 72 hours of dressing application without negative pressure (no negative pressure wound therapy [NPWT]), continuous NPWT, intermittent pressure therapy (IPT) or variable pressure therapy (VPT), using either gauze or foam. Results are shown as means ± SEM of eight experiments.

Intermittent pressure therapy and VPT resulted in more granulation tissue than continuous NPWT (Fig 4). Furthermore, leukocyte infiltration and tissue disorganization, that is, the disruption of the contact between the cells and differences in cell size, were more prominent after IPT and VPT than after continuous NPWT (Fig 5). This wound bed reaction was greater with foam than with gauze under continuous NPWT. When IPT or VPT was applied, the wound bed reaction increased with both wound fillers. For example, the quantity of granulation tissue under foam after treatment with continuous NPWT was similar to that under gauze with VPT. See Figures 4 and 5 for detailed results.

### Tissue ingrowth and force required to remove the wound filler

**Figure 6:**
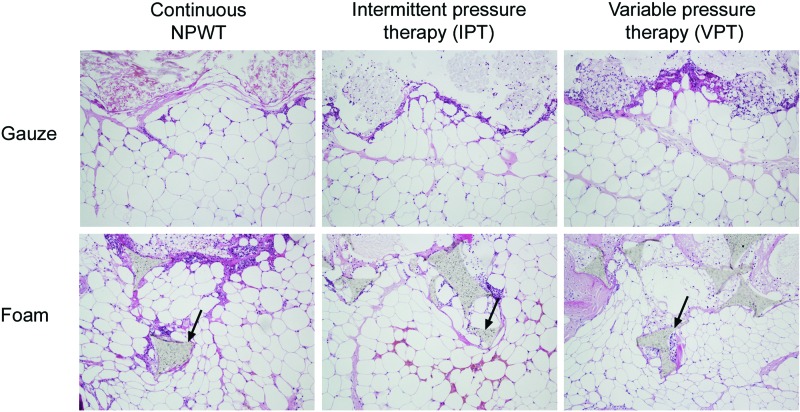
Hematoxylin-eosin stained sections of biopsies from the wound bed with the overlying wound filler, gauze (upper images) and foam (lower images), after 72 hours of continuous negative pressure wound therapy [NPWT], intermittent pressure therapy (IPT), or variable pressure therapy (VPT), using gauze or foam. Note the tissue ingrowth into the foam (indicated by arrows) but not into gauze, regardless of the mode of negative pressure application.

**Figure 7:**
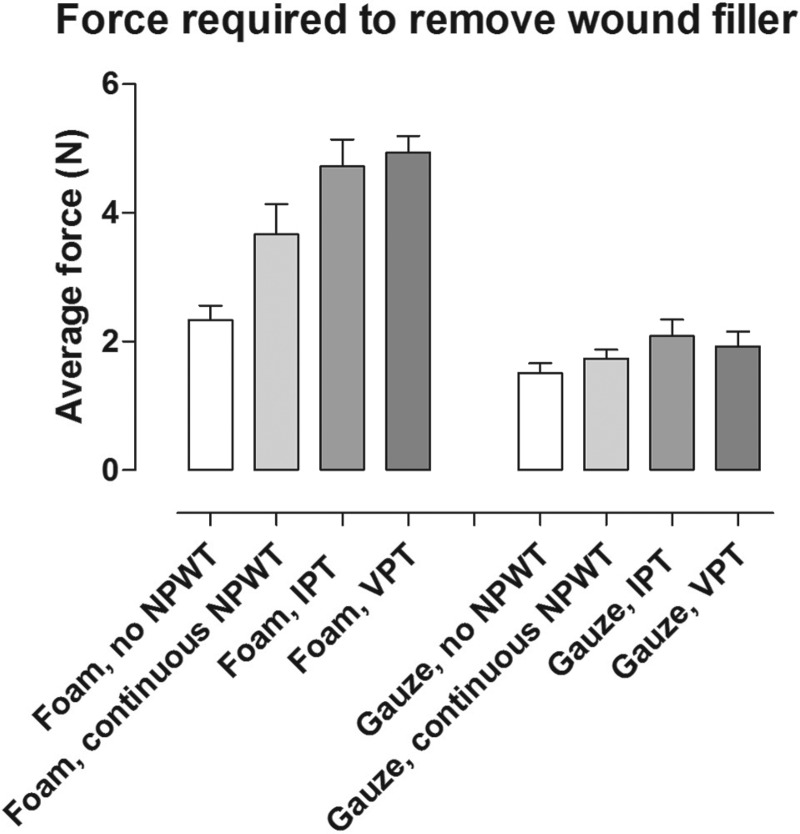
Force required to remove foam and gauze from a porcine wound after 72 hours of dressing application without negative pressure (no negative pressure wound therapy [NPWT]), continuous NPWT, intermittent pressure therapy (IPT), or variable pressure therapy (VPT). The force was plotted as a function of time, and the average force was calculated. The results are shown as means ± SEM of 8 experiments. Note that more force is needed to remove foam than gauze.

Granulation tissue grew into foam but not into gauze, regardless of the mode of negative pressure application. See Figure 6 for representative examples. Greater force was needed to remove foam than gauze from the wound bed tissue after treatment with negative pressure. For gauze, the force was not dependent on the mode of negative pressure application, while for foam, a higher force was needed following IPT and VPT than after continuous NPWT. See Figure 7 for detailed results.

## DISCUSSION

Intermittent pressure therapy has previously been shown to lead to the formation of rapid formation of granulation tissue and accelerated healing. However, treatment has been hampered by the problem of patient pain resulting from the sudden changes in pressure, when going from subatmospheric to atmospheric pressure. Variable pressure therapy is an interesting alternative that ensures smooth transitions between 2 different levels of negative pressure and thus relieve patient pain, while retaining the acceleration in healing. In IPT, the negative pressure is repeatedly switched on and off, while in VPT the pressure is changed cyclically between 2 different levels of negative pressure, thus the negative pressure environment is maintained throughout the therapy. This is a controlled, detailed study of the effects of IPT and VPT on contraction and wound bed morphology.

### Wound contraction

The results of this study show that continuous NPWT induces immediate contraction of the wound, which remains constant during the entire duration of the therapy. Intermittent pressure therapy and VPT also induced immediate wound contraction, but unlike continuous therapy, the contraction gradually increased, that is, the wound area decreased, with the duration of the therapy. After 72 hours, the wounds treated with IPT and VPT were smaller than those treated with continuous NPWT. The reason for this difference cannot be deduced from the present study, but it may be due to the massaging effect of IPT and VPT on the wound edges. This may in turn stimulate tissue remodeling to a greater extent. It is well known that wound contraction results in shearing forces at the wound-dressing interface^7^^-^9 that will affect the cytoskeleton, resulting in a signaling cascade with the release of growth factors, and stimulation of mitosis,^21^^,^^22^ which ultimately leads to granulation tissue formation and accelerated wound healing.^5^ This remodeling of the wound edges may be more pronounced when stimulated by the repeated mechanical effects induced by continuously changing the pressure as in IPT and VPT, than when the level of negative pressure is kept constant, as in continuous NPWT.

### The effects of the negative pressure mode on granulation tissue formation

Intermittent pressure therapy and VPT caused a more pronounced reaction in the wound bed than continuous NPWT. More granulation tissue formation, leukocyte infiltration, and tissue disorganization were seen in the wound bed after IPT and VPT than after continuous NPWT. This suggests faster conversion of the cells to fibroblasts and the laying down of collagen in the process of forming granulation tissue. We speculate that this may be a result of the greater wound contraction observed with IPT and VPT than continuous NPWT, as discussed earlier. Furthermore, our previous results have shown that IPT and VPT have beneficial effects on the blood flow around the wound edges,^15^ including an increase in blood flow 2.5 cm from the wound edge, known to facilitate oxygenation and nutrient supply, accompanied by a decrease in blood flow close to the wound edge (0.5 cm), known to stimulate angiogenesis and granulation tissue formation.

### The effects of the type of wound filler on granulation tissue formation

The choice of dressing used in NPWT has considerable influence on the healing process. The results presented here show a greater wound bed reaction under foam than under gauze. This is in line with previous studies showing that the use of foam as a wound interface in NPWT produces a thick, hypertrophic layer of granulation tissue,^17^^,^^23^ while gauze results in a thinner but denser layer of granulation tissue.^7^^,^^17^ It has been suggested that the dressing should be chosen bearing in mind the wound type for optimal effects. Thick granulation tissue is advantageous for rapid wound healing, but may lead to problems such as fibrosis, scarring, and contractures as the wound heals.^23^ Gauze is often used for NPWT because of its mouldability and ease of application. Gauze has become especially popular among plastic surgeons for wound-bed preparation before grafting.^24^ Gauze may also be a good choice when the cosmetic result is of importance or in cases where scar tissue may restrict movement, for example, over joints. Foam is suitable for wounds that benefit from thick granulation tissue and where scarring does not pose a problem, for example, in sternotomy wounds,^25^ or fasciotomy wounds in upper or lower limb compartment syndrome, where contraction is beneficial,^26^ or in acute wounds with large tissue loss to provide a bridging therapy.^27^^,^^28^

### Clinical implications

We have shown in this study that wound healing can be influenced not only by the choice of dressing but also by the mode of application of negative pressure. Foam is the preferred dressing when a large amount of granulation tissue is desired. However, the ingrowth of tissue into the foam leads to problems when changing the dressing.^19^ There is no ingrowth of tissue into gauze, and we have shown here that it is possible to obtain similar granulation tissue using gauze as with foam, by employing IPT or VPT instead of continuous negative pressure. Using gauze together with IPT or VPT may eliminate the problems associated with ingrowth into foam, while allowing the rapid formation of granulation tissue. The results from the present study support tailoring the amount or characteristics of granulation tissue formation by choosing the mode of NPWT application, that is, IPT, VPT, or continuous NPWT.

### Conclusions

The most common mode of application of negative pressure is the continuous mode, despite the fact that the positive effects of IPT on healing are well documented. The reason for this is the problem of pain often suffered by the patient during IPT. Variable pressure therapy is less painful because it ensures smooth transitions between 2 different levels of negative pressure. The results of the present study show that IPT and VPT perform similarly, in that they result in greater granulation tissue formation than continuous NPWT. This may be the effect of repeated mechanical stimulation, leading to faster tissue remodeling and better blood flow in the wound edges. We propose that the NPWT mode be tailored to the individual wound for optimal effects. One way to tailor the amount or characteristics of granulation tissue formed under NPWT is to choose between foam and gauze, where foam produces a thick granulation tissue but with the drawback of ingrowth, while gauze produces a thinner granulation tissue devoid ingrowth. The results from the present study support tailoring the amount or characteristics of granulation tissue formation by choosing the mode of NPWT application, that is, IPT, VPT, or continuous NPWT. Both IPT and VPT result in rapid granulation tissue formation when using gauze, without the problems associated with ingrowth, as is the case with foam.

## Acknowledgments

This study was supported by Prospera, the Swedish Medical Research Council, Lund University Faculty of Medicine, the Swedish Government Grant for Clinical Research, Lund University Hospital Research Grants, the Swedish Medical Association, the Royal Physiographic Society in Lund, the Åke Wiberg Foundation, the Anders Otto Swärd Foundation/Ulrika Eklund Foundation, the Magn Bergvall Foundation, the Crafoord Foundation, the Anna-Lisa and Sven-Erik Nilsson Foundation, the Jeansson Foundation, the Swedish Heart-Lung Foundation, Anna and Edvin Berger's Foundation, the Märta Lundqvist Foundation, and Lars Hierta's Memorial Foundation.

## References

[1] Argenta LC, Morykwas MJ (1997). Vacuum-assisted closure: a new method for wound control and treatment: clinical experience. Ann Plast Surg.

[2] Banwell PE, Teot L (2003). Topical negative pressure (TNP): the evolution of a novel wound therapy. J Wound Care.

[3] Banwell PE (1999). Topical negative pressure therapy in wound care. J Wound Care.

[4] Morykwas MJ, Argenta LC, Shelton-Brown EI (1997). Vacuum-assisted closure: a new method for wound control and treatment: animal studies and basic foundation. Ann Plast Surg.

[5] Morykwas MJ, Simpson J, Punger K (2006). Vacuum-assisted closure: state of basic research and physiologic foundation. Plast Reconstr Surg.

[6] Lu X, Chen S, Li X (2003). The experimental study of the effects of vacuum-assisted closure on edema and vessel permeability of the wound. Chin J Clin Rehabil.

[7] Borgquist O, Ingemansson R, Malmsjö M (2010). Micro- and macromechanical effects on the wound bed by negative pressure wound therapy using gauze and foam. Ann Plast Surg.

[8] Malmsjo M, Ingemansson R, Martin R (2009). Negative-pressure wound therapy using gauze or open-cell polyurethane foam: similar early effects on pressure transduction and tissue contraction in an experimental porcine wound model. Wound Repair Regen.

[9] Saxena V, Hwang CW, Huang S (2004). Vacuum-assisted closure: microdeformations of wounds and cell proliferation. Plast Reconstr Surg.

[10] Borgquist O, Ingemansson R, Malmsjo M (2010). Wound edge microvascular blood flow during negative-pressure wound therapy: examining the effects of pressures from -10 to -175 mm Hg. Plast Reconstr Surg.

[11] Malmsjo M, Ingemansson R, Martin R (2009). Wound edge microvascular blood flow: effects of negative pressure wound therapy using gauze or polyurethane foam. Ann Plast Surg.

[12] Greene AK, Puder M, Roy R (2006). Microdeformational wound therapy: effects on angiogenesis and matrix metalloproteinases in chronic wounds of 3 debilitated patients. Ann Plast Surg.

[13] Chen SZ, Li J, Li XY (2005). Effects of vacuum-assisted closure on wound microcirculation: an experimental study. Asian J Surg.

[14] Ahearn C (2009). Intermittent negative pressure wound therapy and lower negative pressures—exploring the disparity between science and current practice: a review of the literature. Ostomy Wound Manage.

[15] Borgquist O, Ingemansson R, Malmsjo M (2010). The effect of intermittent and variable negative pressure wound therapy on wound edge microvascular blood flow. Ostomy Wound Manage.

[16] Demaria MG, Stanley BJ, Hauptman JG (2009). Comparison of foam and gauze based negative pressure wound therapy on the healing of open wounds in dogs. Poster Presented at: Clinical Symposium on Advances in Skin & Wound Care; October 22–25.

[17] Borgquist O, Gustafsson L, Ingemansson R (2009). Tissue ingrowth into foam but not into gauze during negative pressure wound therapy. Wounds.

[18] Morykwas M (2003). Sub-Atmospheric Pressure Therapy: Research Evidence. 1st International Topical Negative Pressure Therapy ETRS Focus Group Meeting.

[19] Malmsjö M, Ingemansson R (2010). Tissue trauma and pain during NPWT and wound filler removal (foam and gauze)—examined by immunohistochemistry for substance P and CGRP. Abstract presented at: the Symposium on Advanced Wound Care and the Wound Healing Society Meeting; April 17–20.

[20] Krasner DL (2002). Managing wound pain in patients with vacuum-assisted closure devices. Ostomy Wound Manage.

[21] Austad ED, Thomas SB, Pasyk K (1986). Tissue expansion: dividend or loan?. Plast Reconstr Surg.

[22] Olenius M, Dalsgaard CJ, Wickman M (1993). Mitotic activity in expanded human skin. Plast Reconstr Surg.

[23] Fraccalvieri M, Zingarelli E, Ruka E (2011). Negative Pressure Wound Therapy (NPWT) using gauze and foam: histological, immuno-histochemical and ultrasonography morphological analysis of the granulation tissue and scar tissue. Preliminary report of a clinical study. Int Wound J.

[24] Malmsjö M, Borgquist O (2010). NPWT settings and dressing choices made easy. Wounds Int.

[25] Gustafsson RI, Sjogren J, Ingemansson R (2003). Deep sternal wound infection: a sternal-sparing technique with vacuum-assisted closure therapy. Ann Thorac Surg.

[26] Zannis J, Angobaldo J, Marks M (2009). Comparison of fasciotomy wound closures using traditional dressing changes and the vacuum-assisted closure device. Ann Plast Surg.

[27] Bollero D, Carnino R, Risso D (2007). Acute complex traumas of the lower limbs: a modern reconstructive approach with negative pressure therapy. Wound Repair Regen.

[28] Stannard JP, Robinson JT, Anderson ER (2006). Negative pressure wound therapy to treat hematomas and surgical incisions following high-energy trauma. J Trauma.

